# In vitro studies of the antibacterial activity of *Copaifera* spp. oleoresins, sodium hypochlorite, and peracetic acid against clinical and environmental isolates recovered from a hemodialysis unit

**DOI:** 10.1186/s13756-018-0307-3

**Published:** 2018-01-24

**Authors:** Rosimara Gonçalves Leite Vieira, Thaís da Silva Moraes, Larissa de Oliveira Silva, Thamires Chiquini Bianchi, Rodrigo Cassio Sola Veneziani, Sérgio Ricardo Ambrósio, Jairo Kenupp Bastos, Regina Helena Pires, Carlos Henrique Gomes Martins

**Affiliations:** 1 Nucleus of Research in Sciences and Technology, Laboratory of Research in Applied Microbiology, University of Franca, Avenida Armando Salles de Oliveira, 201, Franca - São Paulo, 14404-600 Brazil; 2 School of Pharmaceutical Sciences of Ribeirão Preto, University of São Paulo, Avenida do Café, s/n, Ribeirão Preto - São Paulo, 14040-903 Brazil

**Keywords:** *Copaifera*, Antibacterial activity, Hemodialysis water, Biofilm, Peracetic acid, Sodium hypochlorite

## Abstract

**Background:**

Patients submitted to hemodialysis therapy are more susceptible to infection, especially to infection by Gram-positive bacteria. Various research works have attempted to discover new antimicrobial agents from plant extracts and other natural products.

**Methods:**

The present study aimed to assess the antibacterial activities of *Copaifera duckei*, *C. reticulata*, and *C. oblongifolia* oleoresins; sodium hypochlorite; and peracetic acid against clinical and environmental isolates recovered from a Hemodialysis Unit. The Minimum Inhibitory Concentration and the Fractionated Inhibitory Concentration Index were determined; the ability of the tested compounds/extracts to inhibit biofilm formation was evaluated by calculating the MICB_50_ and IC_50_.

**Results:**

*C. duckei* was the most efficient among the assayed *Copaifera* species, and its oleoresin was more effective than peracetic acid and sodium hypochlorite. *Copaifera* oleoresins and disinfectants did not act synergistically at any of the tested combinations. Certain of *C. duckei* oleoresin, peracetic acid, and sodium hypochlorite concentrations inhibited biofilm formation and eradicated 50% of the biofilm population.

**Conclusion:**

*C. duckei* oleoresin is a potential candidate for disinfectant formulations. Based on these results and given the high incidence of multi-resistant bacteria in hemodialysis patients, it is imperative that new potential antibacterial agents like *C. duckei* oleoresin, which is active against *Staphylococcus*, be included in disinfectant formulations.

## Background

During End Stage Renal Disease (ESRD), characterized by progressive kidney function loss, the glomerular filtration rate is below 15 mL/min/1.73 m^2^. The kidney can no longer regulate the internal environment, and the patient requires support therapies like hemodialysis, peritoneal dialysis, and kidney transplantation to sustain life. Such support therapies are denominated Renal Replacement Therapies (RRT), being hemodialysis the most widely applied RRT [1–3].

Pontoriero et al. [4] have reported that hemodialysis patients are exposed to 400 L of water used to produce dialysis fluids every week. Despite interposition of a semi-permeable artificial membrane, this water comes into direct contact with the bloodstream. Therefore, knowing and monitoring the dialysis water chemical and microbiological purity is important.

Although dialysis fluid quality depends on a complex chain of devices and procedures and on the implemented quality control procedure, the best strategy to ensure patient safety is to prevent contamination in each dialysis process phase. Water constitutes 95% of the dialysate, and tubes, tanks, and taps represent potential reservoirs for microorganisms to form biofilms, which are extremely hard to eradicate by chemical or mechanical means [5, 6].

Several procedures including physical, chemical, and physicochemical treatments are routinely used to disinfect hemodialysis monitors and the water treatment system [7]. Sodium hypochlorite and peracetic acid disinfectants are commonly applied during disinfection.

Antiseptics and disinfectants play an important role in controlling infection because they act to minimize the spread of microorganisms. However, continued use of these products in hospitals and other health services can trigger bacterial resistance, contributing to antimicrobial resistance development.

Given that bacterial resistance and the risks associated with the use of disinfectants pose a constant challenge, the search for new compounds with antibacterial activity and the development of new products with disinfectant action are crucial.

Effective medicinal plant use has contributed to disseminating information about their therapeutic importance and medicinal effects, validating therapeutic knowledge that has been accumulated for centuries. Nevertheless, the chemical constituents of medicinal plants are not yet fully known [8–12]. Identifying the active components of these plants should increase current knowledge about this inexhaustible natural source of medicinal compounds [13].

The economic and ecological relevance of the species belonging to the genus *Copaifera* has aroused researchers’ interest. According to Leandro et al. [14] and Veiga Jr. and Pinto [15], *Copaifera* oleoresins contain mainly sesquiterpenes and diterpenes. The sesquiterpenes α-copaene, β-caryophyllene, β-bisabolene, α- and β-selinene, α-humulene, and δ- and γ-cadinene are worthy of note.

In vivo and in vitro evaluation has demonstrated that oils obtained from various *Copaifera* species have anti-inflammatory, healing, antiedematogenic, antitumor, trypanocidal, and bactericidal activities [16, 17].

Investigating natural products is clearly essential to the search for new molecules with antibacterial activity. In this sense, this work shall significantly contribute to research into the potential use of *Copaifera* species oleoresins against bacterial strains involved in hemodialysis.

## Methods

### *Copaifera* species oleoresins: Collection and chemical characterization

**Fig. 1:**
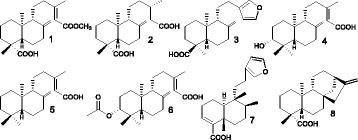
Chemical structures of the compounds identified in *Copaifera* oleoresins: **1**- *ent*-agathic-15-methyl ester (*C. duckei* and *C. reticulata*); **2**- dihydro-ent-agathic acid (*C. duckei*); **3**- *ent*-polyalthic acid (*C. duckei* and *C. reticulata*); **4**- *ent*-3β-hydroxy-copalic acid (*C. multijuga*); **5**- *ent*-copalic acid (*C. multijuga, C reticulata and C. oblongifolia*); **6**- *ent*-3β-acetoxy copalic acid (*C. multijuga*); **7**- Hardwickiic acid (*C. oblongifolia*); and **8**- *ent*-kaurenoic acid (*C. oblongifolia*)

*C. duckei* and *C. reticulata* oleoresins were both collected in Mosqueiro and Brasil Novo, respectively, located in Pará State, Brazil. Plant materials were identified by Silvane Tavares Rodrigues at “Herbário da Embrapa de Belém”, where the voucher specimens NID:96/2012 and NID:03/2013 were stored. *C. oblongifolia* oleoresin was collected in Pirajuba, Minas Gerais State, Brazil. A voucher specimen (NID 14437) was identified by Prof. Dr. Milton Groppo Júnior and deposited at the SPFR herbarium (Faculdade de Filosofia, Ciências e Letras-USP). Chemical characterization of these oleoresins was carried out by HPLC-MS/MS (Fig. 1) and had been previously reported by Santiago et al. [18], Bardaji et al. [19], and Moraes et al. [20]. Collection of the oleoresins used in this study was authorized by the Environment Ministry (MMA, Brazil) and the Chico Mendes Institute for Biodiversity Conservation (ICMBio, Brazil), Number: 35,143–3.

### Bacteria used in the antibacterial assays

The bacteria used during the antibacterial assays had been previously obtained by the research group working in the Applied Microbiology Research Laboratory (LaPeMA) of the University of Franca. The bacteria had been recovered from the hydraulic system (environmental isolates) and from patients (clinical isolates) of a hospital hemodialysis unit and transported to LaPeMA for further isolation in the appropriate culture medium and identification by the commercial identification BBL Crystal Identification Systems (Becton & Dickinson, Sparks, MD, USA).

After identification, the microorganisms were kept in Brain Heart Infusion broth (BHI) containing glycerol at 20% (*v*/v), under cryopreservation (− 80 °C). The following microorganism isolates were used: twelve *Pseudomonas aeruginosa* isolates (water isolates), ten *Staphylococcus aureus* isolates (five isolates from Continuous Ambulatorial Peritoneal Dialysis (CAPD) dialysate, three isolates from hemoculture, one isolate from ascetic fluid, and one isolate from peritoneal liquid), three *Escherichia coli* isolates (water isolates), and nine *Staphylococcus epidermidis* isolates (water isolates).

### *Copaifera* species oleoresins: Minimum inhibitory concentration (MIC) determination

The microdilution method recommended by the Clinical and Laboratory Standards Institute (CLSI) [21], with some modifications, was used to determine MIC (Minimum Inhibitory Concentration). Experiments were conducted in triplicate. The oleoresins were dissolved in dimethylsulfoxide (DMSO; Merck, Darmstadt, HE, Germany) and diluted with Brain Heart Infusion broth (BHI; Difco Labs, Detroit, MI, USA). Then, twelve oleoresin concentrations ranging from 0.195 to 400 μg/mL were tested.

From a stock solution at 3.4%, the disinfectant peracetic acid was diluted in BHI broth and tested at twelve concentrations ranging from 0.000048 to 0.1%. For the disinfectant sodium hypochlorite, a stock solution at 12% was diluted in BHI broth, to obtain twelve concentrations ranging from 0.001464 to 3%.

The inocula were adjusted to give a cell concentration of 5 × 10^5^ CFU/mL [21]. DMSO 5% (*v*/v) was used as negative control, and vancomycin and gentamicin (Sigma) were used as positive control for Gram-positive and Gram-negative bacteria, respectively, from 0.0115 to 5.9 μg/mL. An inoculum was included to monitor the ground for bacterial growth.

The 96-well microtiter plate containing microorganisms was incubated at 37 °C for 24 h in aerobic conditions. After incubation, 30 μL of aqueous resazurin (Sigma) solution at 0.02% was added to each well. Resazurin is an oxireduction probe that allows immediate observation of microbial growth. The blue and red colors represent the absence and the presence of microbial growth, respectively [22].

### Synergistic antimicrobial activity

Checkerboard assays were carried out in triplicate as established by the CLSI [21] to investigate the antimicrobial efficacy of the disinfectants peracetic acid and sodium hypochlorite in association with *C. duckei* oleoresin. Checkerboard assays were performed according to the protocol previously described by Chaturvedi et al. [23]. Synergy tests were conducted in triplicate, and oleoresin and disinfectant concentrations were combined in standard MIC format against 5 × 10^5^ CFU/mL for each bacterium. To evaluate synergism, Fractional Inhibitory Concentration (FIC) index values were calculated as previously established in the literature [23]. Index values were analyzed as follows: FIC index values ≤0.5, > 0.5 to < 1.0, ≥ 1.0 to < 4.0, and ≥4 corresponded to synergistic, additive, indifferent, and antagonistic effects, respectively [24].

### *C. duckei* Oleoresin ability to inhibit biofilm formation as evaluated by the minimum inhibitory concentration of biofilm (MICB_50_)

The Minimum Inhibitory Concentration of Biofilm (MICB_50_) is defined as the minimum antimicrobial agent concentration that can inhibit biofilm formation by 50% or more [25]. MICB_50_ was determined as described in the CLSI guidelines [21] with some modifications. To determine *C. duckei* oleoresin MICB_50_, serial twofold dilutions were prepared in a 96-well polystyrene tissue culture plate (TPP, Trasadingen, Switzerland) containing BHI broth as described previously. The final *C. duckei* oleoresin concentrations ranged from 0.195 to 400 μg/mL. Vancomycin and gentamicin (Sigma) at concentrations between 0.0115 and 5.9 μg/mL were the positive control. The disinfectants peracetic acid and hypochlorite were tested at concentrations ranging from 0.48 to 1000 μg/mL and from 58.59 to 120,000 μg/mL, respectively. Bacterial strains in the absence of antibacterial agent were used as negative controls, and inocula were adjusted to give a cell concentration of 1 × 10^6^ CFU/mL for each bacterium evaluated in the assay. The well contents were discarded after incubation at 37 °C for 24 h. Then, each well was washed three times with 150 μL of sterile Milli Q water and fixed with 150 μL of methanol for 20 min, and MICB_50_ was determined in triplicate by optical density (OD) and by counting the number of microorganisms.

OD measurements aided biofilm formation quantification as described by Stepanovic et al. [26]. Briefly, 150 μL of crystal violet at 2% was added to the microtiter plate wells. After 15 min at room temperature, excess dye was removed by rinsing with tap water, which was followed by air-drying at room temperature. Then, 150 μL of glacial acetic acid at 33% was gently added to each well, to re-solubilize the dye bound to the cells. The microtiter plate was covered with the lid and kept at room temperature for at least 30 min, to minimize evaporation. The OD of each well was measured at 595 nm by using a microtiter plate reader. The percentage of inhibition was calculated by using the equation [25]:

$$ \left(1-{\mathrm{At}}_{595}/{\mathrm{Ac}}_{595}\right)\ \mathrm{x}\ 100, $$

where At_595nm_ and Ac_595nm_ are the absorbance values of the wells treated with *C. duckei* oleoresin and the control, respectively.

Antibiofilm activity was also measured by counting the number of microorganisms. Procedures were the same as the ones described above by Caetano da Silva et al. [27] and Moraes et al. [20], but they were conducted on another microplate. Experiments were performed in triplicate for all the assessed bacteria. After incubation, colonies were counted, and results were expressed as Log_10_ (CFU/mL). The best inoculum concentration and incubation time for the antibiofilm activity assay were selected by standardizing biofilm formation (data not shown).

### *C. duckei* Oleoresin antimicrobial activity against pre-formed biofilms

The Minimum Biofilm Eradication Concentration (represented by IC_50_) of *C. duckei* oleoresin, vancomycin, and disinfectants was determined by a microdilution method conducted in a 96-well microtiter plate as described by Polonio et al. [28], with some modifications. Adherent inoculum (1 × 10^6^ CFU/mL) was incubated in a 96-well microtiter plate for 24 h. After incubation, the biofilm was rinsed, to remove the bacteria that did not adhere to the well.

After 24 h of biofilm growth, adherent biofilms were exposed to oleoresin or vancomycin ranging from 0.98 to 2000 μg/mL. The disinfectants peracetic acid and hypochlorite were tested at concentrations ranging from 0.48 to 1000 μg/mL and from 58.59 to 120,000 μg/mL, respectively. Incubation lasted 24 h.

Adherent bacteria were released and counted, and the percent killing of adherent bacteria was expressed as Log_10_ CFU/mL. Parallel assays were performed against adherent standard inoculum.

The effective concentration that inhibited growth by 50% (IC_50_) was calculated with the GraphPad Prism 5.0 software. All the tests were carried out in triplicate.

## Results

### *Copaifera* species oleoresins: Minimum inhibitory concentration (MIC) determination

As reported previously, the compounds identified in the *Copaifera* oleoresins were as follows: *C. duckey*: *ent*-agathic-15-methyl ester (1), *ent*-agathic acid (2), and *ent*-polyalthic acid (3); *C. reticulata*: (1), (3), *ent*-copalic acid (4), and 3-(methyl)-5-(2,2,6-trimethyl-6-hydroxy-1-cyclohexyl)-pentanoic acid (5) [18, 19]; and *C. oblongifolia*: (4), Hardwickiic acid (6), and *ent*-kaurenoic acid (7) [20]. The MIC values obtained for the *Copaifera* species oleoresins against the tested bacteria varied from 25 to 400 μg/mL (Table 1).

**Table 1:** Minimum Inhibitory Concentration values (μg/mL) of *Copaifera* species oleoresins and disinfectants against bacterial isolates from a hemodialysis unit

Bacteria	Origin	*C. duckei* Oleoresin	*C. reticulata* Oleoresin	*C.oblongifolia* Oleoresin
*P. aeruginosa* (16)	Environmental-Water	> 400	> 400	> 400
*P. aeruginosa* (17)	Environmental-Water	> 400	> 400	> 400
*P. aeruginosa* (18)	Environmental-Water	> 400	> 400	> 400
*P. aeruginosa* (20)	Environmental-Water	> 400	> 400	> 400
*P. aeruginosa* (21)	Environmental-Water	> 400	> 400	> 400
*P. aeruginosa* (22)	Environmental-Water	> 400	> 400	> 400
*P. aeruginosa* (23)	Environmental-Water	> 400	> 400	> 400
*P. aeruginosa* (24)	Environmental-Water	> 400	> 400	> 400
*P. aeruginosa* (25)	Environmental-Water	> 400	> 400	> 400
*P. aeruginosa* (27)	Environmental-Water	> 400	> 400	> 400
*P. aeruginosa* (28)	Environmental-Water	> 400	> 400	> 400
*S. aureus* (2)	Clinical-CAPD dialysate	50	100	> 400
*S. aureus* (3)	Clinical-CAPD dialysate	50	100	> 400
*S. aureus* (4)	Clinical-Ascetic fluid	100	100	> 400
*S. aureus* (5)	Clínico-Peritoneal fluid	100	200	> 400
*S. aureus* (7)	Clinical-Hemoculture	50	100	> 400
*S. aureus* (9)	Clinical-CAPD dialysate	50	100	> 400
*S. aureus* (10)	Clinical-CAPD dialysate	25	50	400
*S. aureus* (13)	Clinical-Hemoculture	50	50	> 400
*S. aureus* (14)	Clinical-Hemoculture	50	100	> 400
*S. aureus* (15)	Clinical-CAPD dialysate	50	100	> 400
*E. coli* (1)	Environmental-Water	> 40	> 400	> 400
*E. coli* (2)	Environmental-Water	400	> 400	400
*E. coli* (3)	Environmental-Water	> 400	> 400	> 400
*S. epidermidis* (54)	Environmental-Water	400	400	> 400
*S. epidermidis* (57)	Environmental-Water	100	200	> 400
*S. epidermidis* (58)	Environmental-Water	400	400	> 400
*S. epidermidis* (60)	Environmental-Water	> 400	> 400	> 400
*S. epidermidis* (62)	Environmental-Water	400	400	> 400
*S. epidermidis* (66)	Environmental-Water	> 400	400	> 400
*S. epidermidis* (67)	Environmental-Water	400	400	> 400
*S. epidermidis* (68)	Environmental-Water	100	100	> 400
*S. epidermidis* (69)	Environmental-Water	> 400	400	> 400
Bacteria	Origin	Sodium Hypochlorite	Peracetic Acid	Positive Control
*P. aeruginosa* (16)	Environmental-Water	468.75	1.95	0.7375^a^
*P. aeruginosa* (17)	Environmental-Water	468.75	3.90	0.7375^a^
*P. aeruginosa* (18)	Environmental-Water	468.75	1.95	0.7375^a^
*P. aeruginosa* (20)	Environmental-Water	937.50	3.90	0.7375^a^
*P. aeruginosa* (21)	Environmental-Water	937.50	1.95	0.7375^a^
*P. aeruginosa* (22)	Environmental-Water	937.50	3.90	0.7375^a^
*P. aeruginosa* (23)	Environmental-Water	468.75	3.90	0.7375^a^
*P. aeruginosa* (24)	Environmental-Water	937.50	3.90	0.7375^a^
*P. aeruginosa* (25)	Environmental-Water	937.50	3.90	0.7375^a^
*P. aeruginosa* (27)	Environmental-Water	937.50	3.90	0.7375^a^
*P. aeruginosa* (28)	Environmental-Water	468.75	0.97	0.7375^a^
*S. aureus* (2)	Clinical-CAPD dialysate	937.50	7.81	1.475^b^
*S. aureus* (3)	Clinical-CAPD dialysate	937.50	7.81	1.475^b^
*S. aureus* (4)	Clinical-Ascetic fluid	937.50	3.90	1.475^b^
*S. aureus* (5)	Clínico-Peritoneal fluid	937.50	3.90	1.475^b^
*S. aureus* (7)	Clinical-Hemoculture	937.50	3.90	1.475^b^
*S. aureus* (9)	Clinical-CAPD dialysate	937.50	3.90	1.475^b^
*S. aureus* (10)	Clinical-CAPD dialysate	468.75	7.81	1.475^b^
*S. aureus* (13)	Clinical-Hemoculture	937.50	3.90	1.475^b^
*S. aureus* (14)	Clinical-Hemoculture	937.50	3.90	1.475^b^
*S. aureus* (15)	Clinical-CAPD dialysate	937.50	3.90	1.475^b^
*E. coli* (1)	Environmental-Water	937.50	7.81	0.7375^a^
*E. coli* (2)	Environmental-Water	937.50	15.62	2.95^a^
*E. coli* (3)	Environmental-Water	937.50	7.81	2.95^a^
*S. epidermidis* (54)	Environmental-Water	937.50	3.90	0.7375^a^
*S. epidermidis* (57)	Environmental-Water	937.50	7.81	0.7375^a^
*S. epidermidis* (58)	Environmental-Water	937.50	3.90	0.7375^a^
*S. epidermidis* (60)	Environmental-Water	937.50	3.90	0.7375^a^
*S. epidermidis* (62)	Environmental-Water	937.50	3.90	0.7375^a^
*S. epidermidis* (66)	Environmental-Water	937.50	3.90	0.7375^a^
*S. epidermidis* (67)	Environmental-Water	937.50	7.81	0.7375^a^
*S. epidermidis* (68)	Environmental-Water	937.50	7.81	0.7375^a^
*S. epidermidis* (69)	Environmental-Water	937.50	3.90	0.7375^a^

^a^ Gentamicin

^b^ Vancomycin

*CAPD* Continuous Ambulatorial Peritoneal Dialysis

MIC values determined for *C. duckei* oleoresin against the bacteria *P. aeruginosa* (all strains), *E. coli* (strains 1 and 3), and *S. epidermidis* (strains 60, 66, and 69); for *C. reticulata* oleoresin against the bacteria *P. aeruginosa* (all strains), *E. coli* (all strains), and *S. epidermidis* (60); and for *C. oblongifolia* oleoresin against the bacteria *P. aeruginosa* (all strains) and *S. aureus* (strains 2, 3, 4, 5, 7, 9, 13, 14, and 15) revealed lack of antibacterial action: MIC values were greater than 400 μg/mL. In contrast, *C. dukei* oleoresin provided promising results against *S. aureus* (strains 2, 3, 4, 5, 7, 9, 10, 13, 14, and 15) and *S. epidermidis* (strains 57 and 68). As for the control drugs gentamicin and vancomycin, they afforded MIC values of 0.7375 μg/mL against *P. aeruginosa* and *S. epidermidis*, 1.475 μg/mL against *S. aureus*, 2.95 μg/mL against *E. coli* strains 2 and 3, and 0.7375 μg/mL against *E. coli* strain 1 (Table 1). *C. reticulata* oleoresin displayed promising antibacterial activity against *S. epidermidis* strain 68 and all the *S. aureus* strains, except for strain 5 (Table 1).

Peracetic acid at concentrations between 0.97 and 3.90 μg/mL, 3.90 and 7.81 μg/mL, 7.81 and 15.62 μg/mL, and 3.90 a 7.81 μg/mL was active against *P. aeruginosa*, *S. aureus*, *E. coli*, and *S. epidermidis*, respectively (Table 1). *P. aeruginosa* and *E. coli* were the most sensitive and the most resistant to peracetic acid, respectively. All the tested strains were sensitive to peracetic acid at the concentration recommended by the manufacturer (1000 μg/mL, see Table 1). In the case of sodium hypochlorite, concentrations between 468.75 and 937.5 μg/mL were active against *P. aeruginosa* and *S. aureus* strains, while a concentration of 937.5 μg/mL was effective against all *E. coli* and *S. epidermidis* strains (Table 1).

### Synergistic antimicrobial activity

**Fig. 2:**
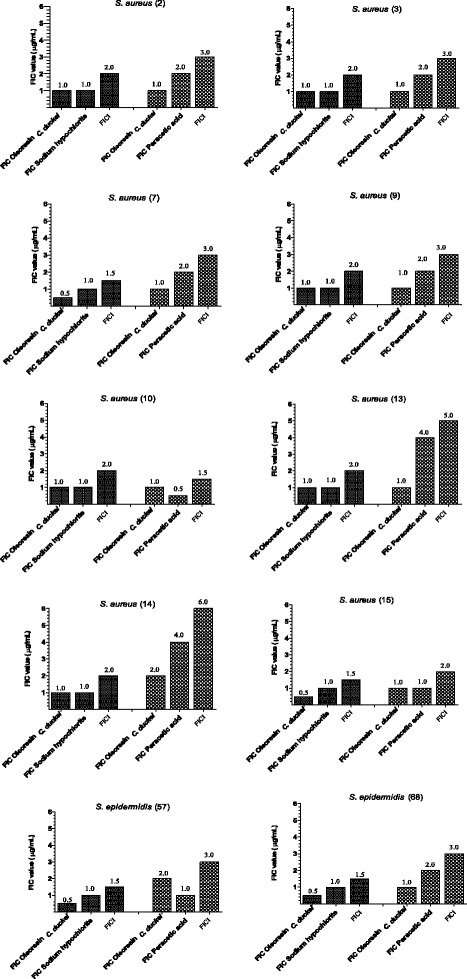
Fractional inhibitory concentration (FIC) and fractional inhibitory concentration index (FICI) of a combination of *C. duckei* oleoresin alone and in combination with disinfectants (sodium hypochlorite or peracetic acid) against bacterial isolates recovered from a hemodialysis unit. FIC assays were performed in triplicate

The present study evaluated *C. duckei* oleoresin combined with the disinfectant peracetic acid against *S. aureus* strains 02, 03, 07, 09, 10, and 15 and *S. epidermidis* strains 57 and 68, to find an indifferent effect. When this combination was tested against *S. aureus* strains 13 and 14, interaction was antagonistic (Fig. 2). As for *C. duckei* oleoresin combined with the disinfectant sodium hypochlorite, interaction was indifferent for all the assessed strains (Fig. 2).

### *C. duckei* Oleoresin ability to inhibit biofilm formation as determined by minimum inhibitory concentration of biofilm (MICB_50_)

On the basis of the results listed in Table 2, *C. duckei* oleoresin had MICB_50_ of 100, 50, 12.5, 6.25, 3.12, and 0.78 μg/mL against *S. aureus* (strain 13), *S. aureus* (strains 7 and 9), *S. epidermidis* (strains 57 and 68), *S. aureus* (strains 2 and 10), *S. aureus* (strain 3), and *S. aureus* (strains 14 and 15), respectively.

**Table 2:** Antibiofilm activities of *C. duckei* oleoresin and disinfectants against bacterial isolates from a hemodialysis unit on the basis of MICB_50_ (μg/mL)

Bacteria	*C. duckei* oleoresin	Peracetic acid	Sodium hypochlorite	Vancomycin
*S. aureus* (2)	6.25	1.95	30,000.0	0.7375
*S. aureus* (3)	3.12	15.62	30,000.0	0.0461
*S. aureus* (7)	50.0	31.25	30,000.0	1.475
*S. aureus* (9)	50.0	1000.0	15,000.0	1.475
*S. aureus* (10)	6.25	15.62	30,000.0	1.475
*S. aureus* (13)	100.0	0.97	15,000.0	1.475
*S. aureus* (14)	0.78	62.5	3.750	0.1844
*S. aureus* (15)	0.78	31.25	15,000	0.0230
*S. epidermidis* (57)	12.5	15.62	1.875	0.7375
*S. epidermidis* (68)	12.5	1000.0	937.5	0.7375

Regarding vancomycin, with standardized MIC values ranging from 0.5 to 2.0 μg/mL [21], results were similar: bacterial bioflm growth inhibition required higher vancomycin concentrations for the strains that were the most resistant to *C. duckei* oleoresin. MICB_50_ varied from 1.475 to 0.0230 μg/mL for vancomycin (Table 2).

Concerning peracetic acid, MICB_50_ was 1.95 and 0.97 μg/mL against *S. aureus* strains 2 and 13, respectively (Table 2), which were approximately 998 and 999 times lower than the concentration recommended by the manufacturer (1000 μg/mL) (Table 2). Among the other strains, the same effect required a range of different concentrations. *S. aureus* strain 9 was the most resistant - peracetic acid MICB_50_ against this strain was 1000 μg/mL (Table 2).

The MICB_50_ values obtained for sodium hypochlorite varied widely: concentrations spanning from 937.5 to 30,000 μg/mL were necessary to achieve the desired effect (Table 2). Hence, biofilm formation inhibition was lower for all the tested bacteria.

*S. aureus* (strains 14 and 15), *S. aureus* (strain 15), *S. aureus* (strain 13), and *S. epidermidis* (strain 68) were the most sensitive to *C. duckei* oleoresin, vancomycin, peracetic acid, and sodium hypochlorite, respectively.

### *C. duckei* Oleoresin antimicrobial activity on pre-formed biofilms

Table 3 lists the results achieved after pre-formed biofilms were exposed to the antimicrobials. The lowest IC_50_ values were 21.85 μg/mL for *C. duckei* oleoresin against *S. aureus* strain 15; 69.85 μg/mL for vancomycin against *S. epidermidis* strain 68; 8.47 μg/mL for peracetic acid gainst *S. epidermidis* strain 68; and 608.7 μg/mL for sodium hypochlorite against *S. aureus* strain 10 (Table 3).

**Table 3:** Antibiofilm activities of *C. duckei* oleoresin, disinfectants, and vancomycin against bacterial isolates from a hemodialysis unit on the basis of IC_50_ (μg/mL), after 24 h of incubation

Bacteria	*C. duckei* oleoresin	Peracetic acid	Sodium hypochlorite	Vancomycin
*S. aureus* (2)	> 2000	65.4	> 120,000	> 2000
*S. aureus* (3)	330.2	480.7	1048	> 2000
*S. aureus* (7)	> 2000	464.6	10,616	> 2000
*S. aureus* (9)	258.6	28.94	> 120,000	> 2000
*S. aureus* (10)	1042	13.85	608.7	> 2000
*S. aureus* (13)	1565	30.84	> 120,000	343.3
*S. aureus* (14)	191.4	213.5	4750	> 2000
*S. aureus* (15)	21.85	56.56	2471	> 2000
*S. epidermidis* (57)	> 2000	8473	910.0	> 2000
*S. epidermidis* (68)	112.7	18.70	896.0	69.85

## Discussion

Given that *Copaifera* oleoresins contain many easily deprotonable acid terpenes [15, 18], the presence of this class of compounds in the oleoresins might contribute to the antibacterial activity observed in this study.

Plants are a source of great chemical and functional diversity, which has allowed investigations into an array of drugs for therapeutic use [29]. *Copaifera* species oleoresins are produced by exudation of trunks from trees belonging to the genus *Copaifera*. Studies have not evidenced that these oleoresins are cytotoxic to mammalian cells, induce behavioral changes, or cause lesions or hemorrhage in the stomach of rats treated with these extracts [30, 31]. Therefore, determining the sensitivity of bacteria, especially Gram-positive organisms, to *Copaifera* species oleoresins could prove a useful tool to combat these microorganisms [32].

Gram-positive bacteria underlie community-acquired infections as well as hospital-acquired infections [33]. Patients undergoing dialysis, mainly through venous access, are at 100 times higher risk of bacteremia than patients that do not require hemodialysis [34]. Most of the times, the etiological agent of such infections is *S. aureus* [35]. In addition, the number of bacteria that are multiresistant to antimicrobial agents like beta-lactam antibiotics, fluoroquinolones, and macrolides has grown at an alarming rate [36]. This has motivated the search for new antimicrobial agents.

Resistance of some of the tested isolates to *C. duckei* oleoresin agrees with the report by Dos Santos et al. [37], who also observed that *E. coli*, *P. aeruginosa*, *S. aureus*, and *S. epidermidis* are resistant to this oleoresin. Moreover, Pacheco et al. [38] evaluated the antimicrobial activities of *Copaifera* species oleoresins against Gram-positive and Gram-negative bacteria, to find that these oleoresins inhibit Gram-positive bacteria at different levels, but they are inactive against Gram-negative bacteria (*E. coli* and *P. aeruginosa*), in agreement with the present study.

Our results are not satisfactory for any of the other tested bacteria and partly agree with the data reported by Santos et al. [39], who evaluated oleoresins obtained from three *Copaifera* species (*C. martii*, *C. officinalis*, and *C. reticulata*) against Gram-positive and Gram-negative bacteria as well as yeasts and dermatophytes. These authors found that *C. martii*, *C. officinalis*, and *C. reticulata* oleoresin concentrations between 31.3 and 62.5 μg/mL inhibit Gram-positive bacteria, including *Bacillus subtilis*, *Enterococcus faecalis*, *S. aureus*, *S. epidermidis*, and methicillin-resistant *Staphylococcus aureus* (MRSA).

Guissoni et al. [40] reported that the oleoresins extracted from two *Copaifera* species, *C. langsdorffii* and *C. reticulata*, can inhibit the growth of *E. coli*, *K. pneumoniae*, *S. marcescens*, *S. aureus*, including *S. aureus* isolates (MRSA). According to these authors, *C. langsdorffii* oleoresin presents MIC of 5000 μg/mL against all the tested bacteria, except for *E. coli*, for which MIC is 620 μg/mL. In the present study, the MIC values determined for *C. reticulata* oleoresin are not satisfactory against any of the assayed bacterial strains.

Papers on the antibacterial activity of *C. oblongifolia* are rare, but *Copaifera* species have been described to be efficient antibacterial agents. Masson et al. [41] assessed the *C. langsdorffii* oleoresin antibacterial activity against standard *S. aureus*, *Streptococcus pyogenes*, *E. faecalis*, *P. aeruginosa*, and *E. coli* strains in vitro, to detect a broad inhibition spectrum for Gram-positive bacteria only. According to these authors, MIC values are 200, 400, and 1100 μg/mL against *S. aureus*, *S. pyogenes*, and *E. faecalis*, respectively. Our MIC values against *S. aureus* are more promising.

In contrast to Gram-negative bacteria, Gram-positive microorganisms are more sensitive to certain compounds. Antimicrobials act on the cell wall, and the different cell wall composition of these classes of bacteria may account for these results. In fact, Gram-positive bacteria present a thick cell wall consisting mainly of peptidoglycan, whereas Gram-negative bacteria display a stratified cell wall consisting of an outer membrane and a thin peptidoglycan layer [42–44]. The unique structural cell wall properties of Gram-negative bacteria may have prevented *Copaifera* species oleoresins from penetrating the cell wall; the external membrane contains lipopolysaccharides, which determine surface properties and alter cell permeability and susceptibility to the investigated oleoresins [45].

Dialysis units have to follow a range of guidelines to disinfect the water distribution system. Among the various disinfecting agents employed in Brazil, sodium hypochlorite and peracetic acid are the most satisfactory [46]. In this context, the present study aimed to evaluate the sodium hypochlorite and peracetic acid activities. Our results corroborate with data from a previous investigation [47] reporting that 58% of the hemodialysis units that conduct disinfection use peracetic acid-based disinfectants. Here, disinfection occurs in 36% of the surveyed units every month, and these units follow the same guidelines followed in the unit where we collected the bacterial isolates for this work.

Compared to the usually recommended concentration of between 25,000 and 45,000 μg/mL [48], we found that all the assayed strains are sensitive to sodium hypochlorite. This has been the standard disinfectant for water treatment and distribution systems in hemodialysis units. Nevertheless, events of bacteremia caused mainly by Gram-negative bacteria in dialysis patients have pointed out that this procedure is inadequate [49–52].

Oliveira et al. [53] tested disinfectants like quaternary ammonium salts; sodium hypochlorite at 0.5%, 1%, and 2%; glutaraldehyde at 2%; Lysoform®; aqueous ethanol solution at 70%; peracetic acid at 2%; and vinegar at 100% against 32 *S. aureus* isolates carried by insects within hospitals, to verify that the bacteria are only resistant to ethanol at 70% and vinegar.

The results we obtained for the assayed *Copaifera* species oleoresins and disinfectants against selected *Staphylococcus* strains show that *Copaifera* oleoresin 100 μg/mL, peracetic acid 7.81 μg/mL, and sodium hypochlorite 937.5 μg/mL inactivate all the bacteria. A comparative analysis reveals that *C. duckei* oleoresin is 12.8 times less efficient than peracetic acid but 9.37 times more efficient than sodium hypochlorite.

Interaction between compounds with antimicrobial activity has been used to reduce minimum inhibitory concentrations and to improve antimicrobial agent efficiency, once participating antimicrobials may act on different bacterial cell sites. Interactions are calculated on the basis of a mathematical equation and are defined as synergistic, additive, indifferent, or antagonist as compared to each isolated antimicrobial MIC [54].

According to the criteria established by Rios and Recio [55] and Gibbons [56], MIC values of 100 μg/mL or lower are promising. Hence, just *C. duckei* oleoresin presents antibacterial potential, and we only assayed this oleoresin in further tests.

The literature does not describe interaction between the *Copaifera* oleoresins and the disinfectants tested herein, but there are some papers on the sysnergism between plant compounds and these same disinfectants against bacteria. One example is the report by Zago et al. [57], who evaluated the synergistic potential of essential oils [(cinnamon (*Cinnamomum zeylanicum Blume Lauraceae*), lemon grass (*Cymbopogon citratus* (DC.) Stapf, Poaceae), peppermint (*Mentha piperita L. Lamiaceae*), ginger (*Zingiber officinale Roscoe Zingiberaceae*), clove (*Caryophillus aromaticus L. Myrtaceae*), and rosemary (*Rosmarinus officinalis L. Lamiaceae*)] combined with eight antimicrobial drugs (chloramphenicol, gentamicin, cefepime, tetracycline, sulfazotrim, cefalotin, ciprofloxacin, and rifampicin) against 12 *S. aureus* strains and 12 *E. coli* human isolates, to demonstrate that *S. aureus* is the most susceptible to interaction between drugs and essential oils, and synergism occurs between lemon grass essential oil and eight of the tested drugs as well as between peppermint essential oil and seven of the tested drugs. In the case of *E. coli*, synergism emerges only between rosemary essential oil and three of the tested drugs and between lemon grass essential oil and two of the tested drugs.

Moraes et al. [20] assessed the synergistic antimicrobial action of *C. oblongifolia* oleoresin with chlorhexidine dihydrochloride against bacteria that cause oral infections. Regarding chlorhexidine dihydrochloride combined with *C. oblongifolia* oleoresin against *S. mutans* (ATCC 25175), *L. casei* (ATCC 11578), *P.gingivalis* (ATCC 33277), and *P. micros* (clinical isolate), the authors found that the effect is indifferent. As for *S. mitis* (ATCC 49456) and *A. actinomycetemcomitans* (ATCC 43717), the effect is additive.

Olmedo et al. [58] examined the synergistic action between sodium hypochlorite and hydrogen peroxide, to observe that the combination of these compounds inactivates planktonic cells and inhibits biofilm formation by *E. coli*, *Salmonella* enterica subsp. enterica, *Klebsiella pneumonia*, and *S. aureus* standard strains as well as *S.* Enterica, *K. oxytoca*, and *E. coli* clinical isolates.

Previous hospitalization, access type during dialysis, comorbidities, gender, time elapsed since the beginning of treatment with dialysis, and previous use of antibiotics contribute to *S. aureus* colonization in dialysis patients [59]. Hence, introducing new compounds with potential antibacterial action in disinfecting solutions is mandatory, especially to combat infection with *S. aureus*.

Despite the development of some research into active combinations of conventional antibiotics, the scientific community has focused on identifying new antibacterial molecules, especially molecules of plant origin, to act as antibiofilm compounds and thus prevent biofilm formation [60].

MICB_50_ is defined as the lowest antibacterial agent concentration that can inhibit biofilm formation by approximately 50% [25]. Moraes et al. [20] investigated *C. oblongifolia* oleoresin antibiofilm activity against bacteria that cause oral infections, to verify that this oleoresin inhibits 50% biofilm formation for the bacteria *Lactobacillus casei* (ATCC 11578) and *Peptostreptococcus micros* (clinical isolate) at 400 μ/mL, *Streptococcus mutans* (ATCC 25175) and *Aggregatibacter actinomycetemcomitans* (ATCC 43717) at 200 μ/mL, and *S. mitis* (ATCC 49456) and *Porphyromonas gingivalis* (ATCC 33277) at 100 μ/mL.

Leandro et al. [61] evaluated the antibiofilm effect of the hydroalcoholic extract of *Copaifera trapezifolia* rich in phenolic compounds against endodontic bacteria, to verify that the oleoresin at 200 μg/mL inhibits *P. gingivalis* (ATCC 33277) and *P. micros* (clinical isolate) biofilm formation by at least 50%.

Alencar et al. [62] reported the *C. langsdorffii* essential oil and oleoresin activities against *Staphylococcus*, *Pseudomonas*, and *Candida* (resistant to azo compounds) and showed that a nanostructured suspension based on *C. langsdorffii* essential oil or oleoresin presents efficient antibiofilm action.

Both sodium hypochlorite and peracetic acid have been tested against *S. aureus*. However, comparison between studies is difficult due to lack of standardized methodologies and concentrations that act on biofilms. Many methodologies have been proposed to probe the microbiocidal action of various disinfectants. Das et al. [63] were one of the first to pioneer the use of microplate methodology to report on the antimicrobial inhibitory effects of certain compounds on planktonic growth and adhered bacteria. These methodologies are based on visual alterations in color or on turbidimetric/colorimetric changes measured by spectrophotometric readings at a specific wavelength. A linear relationship is established between the inoculum size (10–10^7^ CFU/mL) and the exposure time for individual wells, to obtain turbidity between 0.1 and 0.3 OD units within a certain time interval, generally between 1 and 24 h [64]. Several dyes, such as crystal violet, have been proposed to verify microorganism growth after treatment [65]. Another methodological possibility is plating in agar followed by incubation of bacterial inoculum aliquots before and after cell exposure [66, 67].

Svidzinski et al. [68] described that both peracetic acid and sodium hypochlorite at 0.1% (1000 μg/mL) act against MRSA staphylococcus, which agrees with our results demonstrating sodium hypochlorite activity at concentrations as low as 937.5 μg/mL. These authors also found that *S. aureus* strain 15 is approximately 999 times more sensitive to peracetic acid.

Guimarães et al. [69] studied how biocides (hydrogen peroxide at 7% combined with peracetic acid at 0.2%, sodium hypochlorite with 1% active chlorine, ethanol at 70% in aqueous solution and in gel, chlorhexidine digluconate at 0.5%, and povidone iodine at 10%) impact *S. aureus* MRSA biofilm formation, to show that povidone iodine, sodium hypochlorite, and hydrogen peroxide combined with peracetic acid can reduce bacterial film formation by 90%.

Concerning sodium hypochlorite antibacterial activity, our results agree with the data reported by Silva et al. [70], who developed *S. aureus* and *P. aeruginosa* biofilms on polyvinyl chloride (PVC) disks and treated them for 5 min with (a) aqueous chlorhexidine gluconate solution or (b) aqueous sodium hypochlorite solution at 3% and compared them with biofilm growth in buffer solution, to find that all the tested antimicrobials significantly reduce biofilm formation by both microorganisms. Cabeça et al. [71] also evaluated the efficiency of disinfectants like iodine tincture (0.20% *w*/*v*), biguanide (0.50% w/v), quaternary ammonium compounds (0.50% w/v), peracetic acid (0.50% *v*/v), and sodium hypochlorite (1.50% v/v) against planktonic cells (10^8^ CFU/mL) and biofilms formed over sterile stainless steel disks of *Listeria monocytogenes*, *S. aureus*, and *E. coli* reference strains, to verify that planktonic cells of all the organisms are sensitive to all the assayed antimicrobials. Biofim treatment with the disinfectants decreases the number of viable sessile cells. Sodium hypochlorite is the most effective agent, as corroborated by our results.

Toté et al. [72] analyzed 12 disinfectants, including sodium hypochlorite at 1% (10,000 μg/mL) and peracetic acid at 0.3% (3000 μg/mL) diluted in water, against *S. aureus* (ATCC 6538) and *P. aeruginosa* (ATCC 700928) in the planktonic and biofilm growth modes. Samples were treated for 1, 5, 15, 30, and 60 min. The authors found that *P. aeruginosa* planktonic cells are as sensitive as *S. aureus* planktonic cells. Most biocides are effective after 1 min of contact with the microorganisms. Hydrogen peroxide and sodium hypochlorite are the most active biocides against sessile cells: they affect cell viability and diminish biofilm matrix, as also demonstrated herein.

Ueda and Kuwabara [73] investigated *E. coli* O:157, *Salmonella enteritidis*, and *S. aureus* biofilm sensitivities to various disinfectants and sanitizers, to verify that none of the disinfectants (acid, neutral, or alkaline) eliminate sessile cells effectively, all the sanitizers fail to inactivate the biofilm cells completely, and the most effective agent – sodium hypochlorite – still gives colony countings of 25 to 200 microorganisms/mL after treatment at concentrations recommended by the manufacturers. At the studied concentrations, the authors found that bezalkonium chloride, alkyl diaminoethylglycine hydrochloride, chlorhexidine digluconate, and polyhexamethylene biguanide inactivate most of the *E. coli* and *S. enteritidis* cells but not the *S. aureus* cells. The authors concluded that *S. aureus* is the most resistant microorganism evaluated in their study.

Królasik et al. [74] assessed the efficiency of commercially available hydrogen peroxide- and peracetic acid-based disinfecting agents against *Listeria innocua*, *Pseudomonas putida*, *Micrococcus luteus*, and *Staphylococcus hominis* biofilms grown on stainless steel disks, to find that the disinfectants at 0.5% (5000 μg/mL) are ineffective against the bacterial biofilms after 10 min. However, the authors reported that after 30 min at 1% (10,000 μg/mL), *M. luteus* counting reduces by 5 Log UFC/mL in the presence of the disinfectants. Given the results, the concentrations recommended by the manufacturers are ineffective against the assayed bacteria. In our assays, biofilm treatment with the recommended peracetic acid concentration of 0.1% (1000 μg/mL) for 24 or 48 h provides colony countings reduced by 11 Log UFC/mL in the case of the initial inocula of *S. aureus* strains 2, 3, 9, 10, 13, and 15 and *S. epidermidis* strains 57 and 68.

Gilbert et al. [75] simultaneously evaluated five disinfectants diluted in culture medium containing peracetic acid (Proxitane 4002, Solvay Interox Ltd., Warrington, UK) against *E. coli* and *S. epidermidis* planktonic and biofilm cells. Disinfectant concentrations varied from 0 to 100 nanomoles (nmol)/L. Analyses considered the planktonic/biofilm cell ratio corresponding to 95% of dead cells within 30 min of exposure. The authors demonstrated that biofilm age affects results very little, but data heavily depend on the tested microorganism and disinfectant. They also showed that peracetic acid is the most effective agent against planktonic cells and significantly decreases biofilm cell activity at similar concentrations. One of the explanations for data dependence on concentration is that antimicrobial agents, especially agents that interfere in the membrane potential (for example, oxidizing agents such as peracetic acid), operate in many sites and through several mechanisms. Our results agree with the results of Gilbert et al. [75] in the case of planktonic cells. However, we verified that sodium hypochlorite is more efficient against biofilm cells: IC_50_ is 57.425% as compared to 33.060% achieved for peracetic acid.

In conclusion, MIC determination showed that *C. duckei* oleoresin is the most effective among the evaluated *Copaifera* species oleoresins: it inhibits the growth of *S. aureus* and *S. epidermidis* strains. Interaction between *Copaifera* oleoresins and disinfectants does not result in synergism. All the investigated strains form biofilms in the assayed conditions. Overall, the capacity of disinfectants to inhibit biofilm formation varies widely, and very low *C. duckei* oleoresin, vancomycin, peracetic acid, and sodium hypochlorite concentrations are necessary to achieve this effect. On the basis of the Minimum Biofilm Erradication Capacity, represented by IC_50_, sodium hypochlorite is the most effective antimicrobial tested herein.

## Conclusions

In general, *C. duckei* oleoresin is as active as peracetic acid in terms of *S. aureus* biofilm cell eradication. Therefore, this oleoresin is potentially useful in formulations that aim at *S. aureus* disinfection even when the microorganism grows in the biofilm mode, with possible application in dialysis settings.

## Abbreviations

BHI: Brain heart infusion broth
CAPD: Continuous ambulatorial peritoneal dialysis
CLSI: Clinical and laboratory standards institute
FIC: Fractional inhibitory concentration
MIC: Minimum inhibitory concentration
MICB_50_: Minimum inhibitory concentration of biofilm
OD: Optical density
